# Advances in e-learning in undergraduate clinical medicine: a systematic review

**DOI:** 10.1186/s12909-022-03773-1

**Published:** 2022-10-07

**Authors:** T. Delungahawatta, S. S. Dunne, S. Hyde, L. Halpenny, D. McGrath, A. O’Regan, C. P. Dunne

**Affiliations:** 1 School of Medicine, University of Limerick, Limerick, Ireland; 2 Centre for Interventions in Infection, Inflammation & Immunity (4I), University of Limerick, Limerick, Ireland

**Keywords:** e-learning, Online learning, Distance learning, Medical education, Medical students, Clinical medicine, Systematic review

## Abstract

**Background:**

E-learning is recognised as a useful educational tool and is becoming more common in undergraduate medical education. This review aims to examine the scope and impact of e-learning interventions on medical student learning in clinical medicine, in order to aid medical educators when implementing e-learning strategies in programme curricula.

**Methods:**

A systematic review compliant with PRISMA guidelines that appraises study design, setting and population, context and type of evaluations. Specific search terms were used to locate articles across nine databases: MEDLINE/PubMed, ScienceDirect, EMBASE, Cochrane Library, ERIC, Academic Search Complete, CINAHL, Scopus and Google Scholar. Only studies evaluating e-learning interventions in undergraduate clinical medical education between January 1990 and August 2021 were selected. Of the 4,829 papers identified by the search, 42 studies met the inclusion criteria.

**Results:**

The 42 studies included varied in scope, cognitive domain, subject matter, design, quality and evaluation. The most popular approaches involved multimedia platforms (33%) and case-based approaches (26%), were interactive (83%), asynchronous (71%) and accessible from home (83%). Twelve studies (29%) evaluated usability, all of which reported positive feedback. Competence in use of technology, high motivation and an open attitude were key characteristics of successful students and preceptors.

**Conclusions:**

Medical education is evolving consistently to accommodate rapid changes in therapies and procedures. In today’s technologically adept world, e-learning is an effective and convenient pedagogical approach for the teaching of undergraduate clinical medicine.

**Supplementary Information:**

The online version contains supplementary material available at 10.1186/s12909-022-03773-1.

## Background

E-learning, a pedagogical approach supported by the principles of connectivism learning theory, involves the use of technology and electronic media in knowledge transfer [1, 2]. Connectivism views knowledge as a fluid entity circulated through technology enabled networks that foster interactions between individuals, organizations, and societies at large [2]. Based on this conceptual framework, medical curricula can potentially benefit from enhanced communication and knowledge exchange using technology.

Common e-learning instructional designs in clinical medicine include “online and offline computer-based programmes, massive open online courses, virtual reality environments, virtual patients, mobile learning, digital game-based learning and psychomotor skills trainers”[1]. To maximize the potential for e-learning, it seems rational that the roles and needs of the e-learner, e-teacher and host institution should be defined and appreciated. According to the Association for Medical Education in Europe (AMEE), an e-learner is any individual taught in an online learning environment [1]. As the role of the e-learner is central to the learning process, effective e-learning strategies should consider potential learning challenges encountered by the e-learner. Employing skilled e-teachers and providing them with sufficient supports are also important considerations. Furthermore, institutional management of the content versus process elements of educational technology use should best align with the objectives of the program [1]. For example, if the intent is to provide student access to digital content, then managing sound or video files, podcasts, and online access to research papers, clinical protocols, or reference materials, should be prioritized. On the other hand, if the focus is on student participation in digital activities, then managing processes such as discussion boards and test-taking should take precedence. Accounting for the role of the e-learner, e-teacher, and host institution in this manner, can result in successful implementation of an e-learning system. In fact, e-learning has been shown to be at least as effective as, and can serve as an adjunct to, face-to-face teaching and learning methods [3–5].

An institution may choose to employ educational technologies for the entirety of the course or provide a combination of online and in-class interactions, with the latter approach referred to as ‘blended learning’ [1]. Incorporation of e-learning into the curriculum allows for new avenues of interactive knowledge and skill transfer between teachers and students and amongst students. Interactions are not limited to face-to-face conversations but can involve text, audio, images, or video, thereby enriching the learning experience. Giving access to a greater breadth of learning resources further develops lifelong learning skills in students as they are required to independently evaluate and extract the pertinent information [1]. E-learning interventions can also be accessed at any time from almost any location, which facilitates a student-centred approach through self-directed and flexible learning [6]. As such, e-learning is an attractive instructional undergraduate health education approach [7].

To date, e-learning interventions in the sciences, particularly anatomy [8] and physiology [9], and postgraduate medical training [3, 4] have been described. However, their use has not been reviewed systematically in the specific context of augmenting, enhancing or supporting student learning in undergraduate clinical medicine [10], or replacing face-to-face learning with online learning in the case of COVID-19 emergency remote teaching. In 2014, survey responses from senior medical students in Illinois, reported use of online collaborative authoring, multimedia, social-networking, and communication tools as point of opportunity study resources during clinical rotations [11]. Additionally, the COVID-19 pandemic has necessitated stepping away from traditional classroom and bedside teaching, and development of more flexible course delivery. A recent survey by Barton et al. collected 1,626 responses from medical students across 41 medical schools in the United Kingdom during the COVID lockdown. Results of study resources accessed daily showed that 41.6% of students used information provided by university (PowerPoint lecture slides, personal notes), 29.6% accessed free websites and question banks, and 18.4% accessed paid websites and question banks [12]. The work therefore suggests a strong tendency for students to supplement university materials with online resources [12, 13]. The popularity of online learning platforms seems to stem from an association with achieving higher exam scores [14, 15], ability to self-monitor knowledge gaps [16], improved knowledge retention from repeat exposure [17, 18], and to practice exam technique [16].

Medical school educators are, therefore, called to evaluate e-learning approaches and to consider incorporation of suitable strategies into current curricula to ensure equitable access and student success. Thus, we aimed to systematically review the scope and impact of e-learning interventions published regarding undergraduate clinical medicine, and to inform medical educators of the effectiveness and character of various online learning environments.

## Methods

The Preferred Reporting Items for Systematic Reviews and Meta-Analyses (PRISMA) guidelines are used for the reporting of this systematic review [19]. The PRISMA checklist is included as Additional File 1.

### Search methods

The early 1990s marked the commercial availability of computer-based learning multimedia [20] as well as the emergence of online education programs [21]. Thus, medical subject headings (MeSH), key words and specific database headings were used to locate articles published between January 1990 and August 2021: ‘e-learning’ or ‘digital resources’ or ‘internet learning resources’ AND ‘medical education’ AND ‘undergraduate’ AND ‘techniques’ or ‘programmes’ or ‘interventions’. The search was piloted on PubMed and adapted subsequently for the databases. A total of nine databases were searched: MEDLINE/PubMed, ScienceDirect, EMBASE, Cochrane Library, ERIC, Academic Search Complete, CINAHL, Scopus, Google Scholar and grey literature. The bibliographies of each selected paper were searched manually for further studies. Websites of medical education organisations were searched for position statements and guidelines, including the Association for the Study of Medical Education, AMEE and the British Medical Journal.

### Inclusion and exclusion criteria

Only studies in the English language that evaluated an e-learning intervention in subjects related to clinical medicine were selected. These included: family medicine, surgery, internal medicine, radiology, psychiatry, dermatology, paediatrics and obstetrics. Studies that did not involve undergraduate medical students, were based on pre-clinical sciences or were not focussed on an e-learning intervention were excluded. Studies that focussed on the use of internet for assessment and course administration only were not included. Additionally, studies that described interventions but not their evaluation were excluded. Of the 4,829 papers identified by the search, 42 studies were deemed eligible for inclusion in this review.

### Data extraction and analysis

AMEE guidelines on e-learning interventions [1] were used to modify a previous data extraction tool that had been used in a systematic evaluation of effectiveness of medical education interventions [22]. This was subsequently piloted and refined by three of the authors until consensus was achieved to form the data extraction tool (see Additional File 2). With application of connectivism, individual elements of e-learning were identified to infer and appreciate their collective effects on the learning process. More specifically, data was extracted by examining two central questions: *how* and *when* to use e-learning in undergraduate clinical medical education. The primary outcomes relating to *how* to use e-learning were: instructional features that made the e-learning intervention effective; usability features; assessment of effectiveness and quality of the intervention. Primary outcomes relating to *when* were: the context, and the learner and preceptor characteristics. In addition to the outcomes measured, descriptive data was also extracted to summarise the studies including: the study design, setting and population; context and discipline; type of evaluations. All selected papers were filed in an Endnote library and the data extraction tool for each was stored in an Excel file, a summary of which is provided as Additional File 2 and Additional File 3.

Guidelines for evaluating papers on medical education interventions from the Education Group for Guidelines on Evaluation were used as a framework to assign a global score for the strength of each paper [23]. Among these guidelines, significant value is placed on development of strong intervention rationale and intervention evaluation methods [23]. The impact of the evaluation was also measured using Kirkpatrick’s levels, a recognised system of understanding the effect of interventions [24]. The first level, reaction, is a measure of learner satisfaction with the intervention [24]. The second Kirkpatrick level, learning, is a measure of change in knowledge, skills, or experience. The third Kirkpatrick level of behaviour is a measure of behavioural change. The final level, results, is a measure of overall impact on the organization (i.e., improved quality of work, reduction in time wasted, better patient care).

## Results

### Search results

**Fig. 1:**
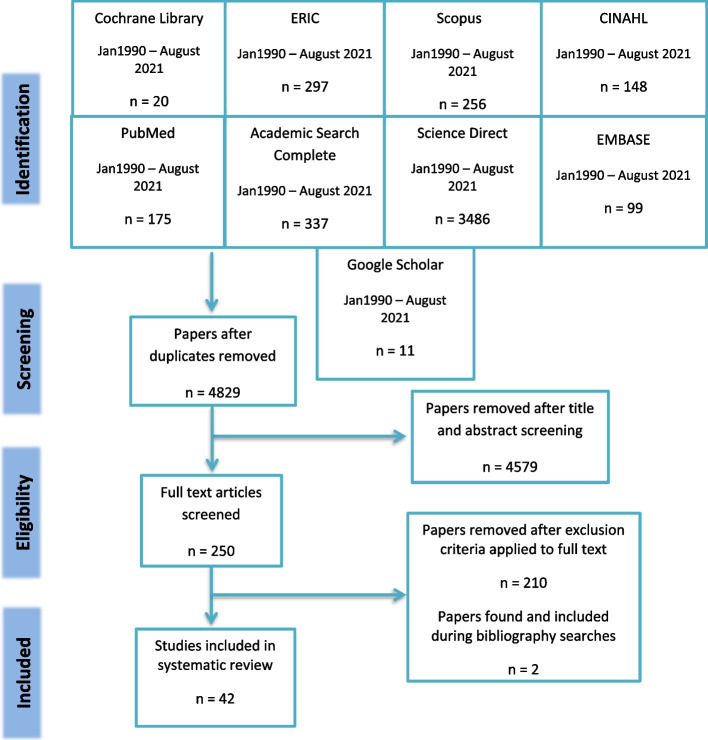
PRISMA flow diagram

A total of 4,829 papers were retrieved from database and manual searches, and this number was reduced to 42 after removal of duplicates and application of inclusion/exclusion criteria at set stages (see Fig. 1 for the PRISMA flow diagram). Two papers were retrieved from manual searches of bibliographies [25, 26]. The main reasons for excluding studies were a lack of focus on undergraduate medical students (112 studies) or absence of an e-learning intervention (34 studies).

### Design of included studies

The year of publication ranged from 2003 to 2021, with most conducted within the past ten years (31 studies). Interventions were conducted in nine different countries, mainly the United States (13 studies) and Germany (9 studies). More than half of the studies were conducted in the European Union (21 studies). Several research designs were described, including 17 observational studies [25, 27–42], 13 randomised control trials [26, 43–54], three non-randomised control trials [55–57], eight qualitative studies [58–65], and one mixed methods study [66]. Thirteen of the total studies included data collection both pre- and post- intervention [25, 27, 31, 34, 36, 38, 39, 45, 48, 52–54, 61]. Six studies had follow-up data (collected weeks to months after intervention) [34, 45, 49, 52, 54, 56] and twelve papers reported ethical approval [28–31, 33, 34, 39, 40, 42, 46, 49, 54]. Furthermore, eight studies described learning theories in the development or evaluation of medical curricula [29, 30, 33, 49, 51, 52, 56, 58]. Of these studies, five referenced constructivism [29, 49, 51, 52, 58] three studies highlighted cognitivism [30, 56, 59], and one study evaluated behaviourist learning theory [33].

### Study population

**Fig. 2:**
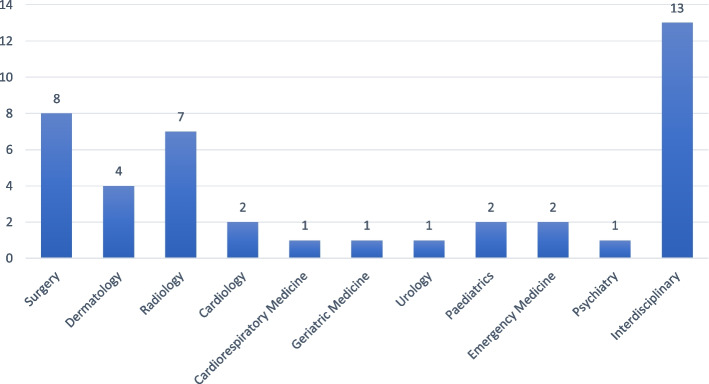
Intervention discipline

Students in the third year of medical school experiencing clinical exposure were the most commonly studied (sixteen studies), with fourteen studies involving multiple cohorts of students (see Additional File 3). Sample sizes ranged from 10 to 42,190 individuals. The most common disciplines investigated were interdisciplinary (13 studies), surgery (8 studies), radiology (7 studies), and dermatology (4 studies) (see Fig. 2 Intervention Discipline).

### Intervention characteristics

**Fig. 3:**
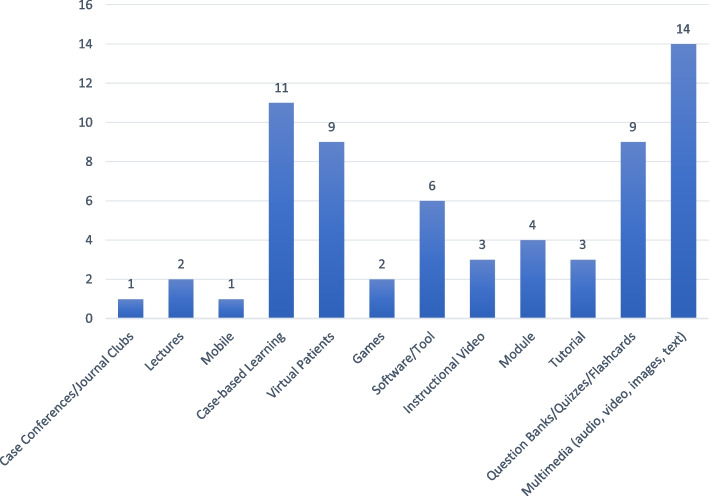
Intervention type

**Fig. 4:**
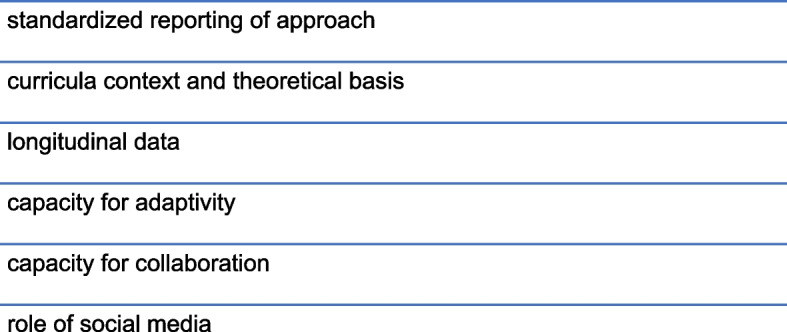
Future intervention design recommendations

Twelve types of intervention were described and the most commonly used were multimedia platforms (fourteen studies) and case-based learning (eleven studies), as per Additional File 2 and Fig. 3. In terms of cognitive domain, 27 interventions were in the domain of knowledge [25–27, 29, 30, 32–35, 39, 40, 42, 43, 47, 48, 50, 52–54, 57, 60–64, 66, 67]; eight were in the domain of skills [9, 30, 31, 36, 37, 46, 49, 51] and seven in combined knowledge and skills [38, 41, 44, 45, 56, 59, 65]. The interventions ranged in duration from a single session to a complete academic year. Thirteen of the interventions were synchronous, where users log on at a given time [8, 26, 27, 31, 33, 34, 37, 43, 47, 51, 52, 58, 66], and the remaining 29 used an asynchronous platform (users logging on independently in their own time). Seven were accessible in a classroom setting only [26, 27, 36, 47, 52, 58, 66] while the others could be accessed from home (Fig. 4).

Reported roles for e-learning within the curriculum included a revision aid for examinations [58]; the flipped classroom concept [44, 57], whereby lectures held after an e-lecture become an interactive session; to facilitate an online community where knowledge could be discussed/ shared [25]; and, enabling just-in-time learning through timely access to facts [30, 31, 37]. Seven (17%) of the 42 interventions were didactic in approach [27, 30, 37, 55, 57, 63, 65], while the others were interactive. Twelve studies described a collaborative approach, whereby students discussed cases and problems with one another and engaged in role-plays [25, 26, 36, 38, 40–42, 46, 52, 59, 61, 66]. The context of e-learning in relation to the curriculum was not stated in ten of the studies but another thirteen studies used the terms “adjunct”, “complement”, “supplement”,”hybrid” and “blended” to illustrate the common theme of integrating e-learning with traditional learning [25, 29, 30, 32, 44–47, 50, 56–58, 62, 63]. Seven studies describe temporary replacement of traditional curricula with e-learning platforms in response to COVID-19 [33, 40–42, 61, 62, 64]. Eight studies described a pilot phase or the inclusion of students in the development of the intervention [33, 37, 44, 45, 48, 49, 53, 66]. Nineteen of the interventions had a built-in assessment, with multiple choice questions being used in most cases, to evaluate whether an improvement in learning had taken place [25, 27, 31, 34, 37, 39, 43, 45–52, 54, 55, 59, 66]. Justification for the chosen assessment strategy or a statement on its suitability was included in two studies [50, 66]. Kourdioukova et al. reported an improvement in knowledge and skills with computer supported collaborative case-based approach as judged by in-built multiple-choice questions (MCQ), suggesting the importance of content-specific scripting [66]. Schneider et al. used a combination of MCQ and survey, and justified their use by demonstrating that learning improved with the intervention compared to the control [50]. Five of the interventions used end of module assessments as the marker of quality [26, 29, 53, 56, 57], with one stating that this was not a suitable mechanism due to its inability to assess the students’ ability to take a patient history or perform a clinical examination [53].

### Intervention evaluation

Each study was given a global rating from 1–5 based on guideline criteria from the Education Group for Guidelines on Evaluation, including whether learning outcomes and curricular context were outlined and the power and rigor of the studies [23] (Additional File 2). Accordingly, eleven studies scored 4/5; two scored 3.5/5; twelve studies scored 3/5; twelve studies scored 2.5/5; and five scored 2/5 (σ = 0.138).

### Intervention effectiveness and acceptability

Nine studies described an impact matching a Kirkpatrick level 1, where the student reaction to e-learning intervention was evaluated using student surveys or questionnaires [32, 35, 44, 58, 60–62, 64, 65]. All these studies report that most students were satisfied with the addition of an e-learning intervention. For instance, Orton et al. note that over 91% of survey responses either ‘strongly agreed’ or ‘agreed’ that use of computer-based virtual patients enabled learning [35].

Twenty-one (50%) of the 42 studies evaluated acceptability [26, 30, 32, 33, 36, 37, 40–42, 44, 48, 53–58, 63, 65, 66, 68]. Of these, 17 reported that the intervention was acceptable. A neutral attitude was reported to a radiology e-learning intervention that involved peer collaboration and was found to be time consuming[66]. Attitude in another study was much more favourable in junior years than in senior years, with the authors commenting on the conflict between completing assignments and preparing for high stakes examinations [55]. Another study that focussed on acceptability, with positive outcomes, found that perceived utility and ease of use were the key factors [30]. Twelve (57%) of the 21 studies further evaluated usability [30, 36, 37, 40–42, 44, 53, 56–58, 65], all with positive outcomes, but only one used a formal usability assessment tool [58]. In that study, Farrimond et al. found that a usable intervention should be: simple and intuitive to use and, from a learner perspective, interactive and enjoyable [58]. In the development of virtual lectures, ease of navigation, audio-visual quality and accessibility were the key usability features [57]. Wahlgren et al. concluded that as well as navigation, interactivity is a priority for e-learning development [53]. Regarding mobile learning, the display should be adaptable to varying screen sizes, termed ‘chunking’, and it should be suitable for a number of platforms [30].

Twenty-nine (69%) of the 42 studies described an impact matching a Kirkpatrick level 2, where evaluation of whether learning took place was assessed through post intervention scores [25, 27, 31, 36, 38, 39, 47, 48, 50, 52–54, 56, 57, 61], final exam results [26, 29, 45, 66], direct observation [28, 31, 33, 43, 46, 51, 55] and student survey [25, 26, 30, 37–42, 45, 48, 49, 53, 54, 56, 65, 66]. Among these studies, two studies had included both pre- and post- intervention evaluations but neither had a control group nor longer term follow-up [25, 27]. One randomised control trial showed a statistically significant improvement in factual knowledge acquisition after participation in an online module as judged based on performance in end of year assessments, compared to a traditional teaching control group (84.8% ± 1.3 vs. 79.5% ± 1.4, *p* = 0.006, effect size 0.67) [26]. Likewise, Davis et al., found that the use of a procedural animation video on mobile device resulted in higher medical student scores on skills checklist (9.33 ± 2.65 vs. 4.52 ± 3.64, *p* < 0.001, effect size 1.5) [30]. Similarly, in Sijstermans et al., mean students’ self-evaluation of their skills using five-point Likert scale questionnaire, before and after two patient stimulations showed improvement (3.91 ± 0.28 vs 3.56 ± 0.34, *P* < 0.0001, effect size 1.12). Furthermore, in one study employing a problem-based e-learning approach, the number of first-class honours awarded were found to be significantly improved when compared to control group [29]. However, in another study using a problem-based e-learning intervention, no significant difference was found between control and intervention groups in subsequent examinations (*p* = 0.11) [53]. In contrast, Al Zahrani et al. found that delivery of new e-learning platforms (Blackboard Collaborate, ZOOM) in response to COVID-19 was poorly accepted by students, whereby 59.2% did not feel adequately educated on learning outcomes, 30% felt no educational difference between e-learning and traditional curriculums, and 56.1% felt e-learning is insufficient as an educational tool for the health sciences [40].

Four studies demonstrated a change in student behaviour in line with Kirkpatrick level 3 [50, 52, 59, 63]. In de Villiers et al., it was found that students were using podcasts to learn course content and the classroom teaching setting to strengthen their understanding, inadvertently accepting the flipped classroom approach [63]. In Sward et al., students who were assigned to a gaming intervention were more willing to engage in answer creating and answer generating as well as independent study of subject materials prior to session time [52]. Similarly, in Schneider et al., students in the computer case-based intervention group were found to invest more time into studying course subjects (38.5 min vs 15.9 min) which resulted in significantly higher test scores [50]. Finally, in Moriates et al., following the integration of value-based modules, students have reported increased awareness of patient needs and discussions with peers regarding value-based decision-making during clerkship [59].

### Learner and preceptor characteristics

Learner characteristics identified to enable successful e-learning include: good digital skills, less resistance to change [32] and a willingness to collaborate with peers [66]. Preceptor characteristics were not described in most of the studies, but the role involved guiding students through their learning [33, 46, 61, 66], selection of topics of broad interest to students [60], technical support [54], student evaluation[28, 31, 37, 40, 42, 45, 46, 49, 51], content development and management [32, 41, 42, 46, 54, 62] and providing feedback and clear instruction on what is expected of the learners [28, 37, 40, 42, 51, 54, 60].

## Discussion

The COVID-19 pandemic resulted in global university closures during periods of lockdown, necessitating educators to quickly adopt alternate pedagogical approaches. As a result, there has been a substantial increase in the use of e-learning, by which teaching and learning activities occur at a distance on online platforms [69].

In enabling a shift in the control of knowledge acquisition and distribution from the teacher to the student, e-learning facilitates the learning process. Learners filter the available information, develop new perspectives, log into networks to share their understanding, and repeat the cycle [2]. This view of learning as a fluid and dynamic process is the basis of the learning theory of connectivism and highlights the benefit of this instructional design in medical education – a field amenable to rapid changes in therapies and procedures. In fact, educational theorists have significantly influenced the development of medical curricula throughout history. Amongst the 25 higher impact studies (achieving a global score greater or equal to 3), only 7 studies (28%) were found to have described theoretical underpinnings [30, 33, 49, 51, 52, 58, 59]. Initially, the behaviourist perspective supported pedological practices [70]. Behaviourism described learning as largely deriving from responses to external stimuli and led to curricula aimed to influence behaviour through reward and positive and negative reinforcement. In one study reviewed, the lack of direct observation of non-verbal communication by instructors was seen as a significant learning challenge in the virtual environment [33]. A shift from behaviourism to cognitivism later ensued with the belief that the brain is much more than a ‘black box’ and learning rather involved mental processing and organization of knowledge, and memory functions [70]. With the recognition of individual differences in the learning process, online systems attempted to introduce interventions that suited multiple learning strategies. For example, learning from auditory narration with animation was found to be more effective than use of text with animation [71]. This review further highlighted the impact of repetition [30] and clinical reasoning [56, 59] on the learning process. More recently, constructivist learning theory and the perception that learners incorporate new information into pre-existing knowledge schemas has greatly contributed to reformation of medical education [70]. Incorporating real world connections [29, 49, 58], building on motivations [52], application of feedback [51] and continuous reflection [49] has been noted in this review as important factors in knowledge handling and retention. Presently, e-learning interventions often utilize aspects of more than one theoretical perspective. For instance, problem-based learning interventions have emphasised the critical thinking processes of cognitivism and the self-direction of constructivism [29]. While primary studies have increased the reporting of underlying theory over time, there is still a significant lack of discussion – future work should reference theoretical principles to objectively frame and assess online education.

In addition to recognizing the needs of the e-learner, identifying required skills of e-teachers and developing content that appropriately supplement the curriculum are vital to ensuring successful implementation of an e-learning system [1]. Therefore, this study involved review of studies published between 1990 and 2021, assessing the effectiveness and character of various online learning environments in undergraduate clinical medical education. Specifically, these studies involved medical students pursuing medicine as a primary degree and those enrolled with prior degrees.

### Intervention design

Critical appraisal of the collected studies using EGGE criteria, identified seventeen studies (40%) meeting a global rating of less than 3. The EGGE criteria encompass a standardized framework by which quality indicators can be recognized. Lower ratings of included studies suggests that conducting and reporting of e-learning interventions is largely lacking in methodological rigour and therefore limits transferability of study results. This finding is consistent with conclusions from a review by Kim et al., describing how most of the existing literature on e-learning interventions have little quantitative data, evaluate a limited range of outcomes and have significant gaps in study designs [72]. Additionally, only 13 (31%) randomized control trials (RCTs) were included in the review [26, 43–54]. Amongst these studies, five reported pre and post test scores [45, 48, 52–54], three of which report long term follow up [45, 52, 54]. Interestingly, all the RCTs report no significant differences in knowledge mastery between control and intervention groups. However, in the immediate short term, e-learning interventions were associated with greater learner satisfaction. For example, in Lee et al., mobile learning with interactive multimedia had higher satisfaction scores compared with conventional Microsoft PowerPoint Show content, despite non-significant differences in knowledge gain [48]. Similarly, in the study by Wahlgren et al., the majority of students in the intervention group reported that the interactive computerised cases enabled better understanding of disease diagnosis and management, particularly referencing the user-friendliness and feedback [53]. Yet, knowledge gain as assessed by post-intervention examination scores did not show statistically significant differences between the two groups. Systematic reviews examining the effect of e-learning on nursing education have also demonstrated no differences between e-learning and traditional teaching modalities but report high satisfaction rates with the former [73, 74]. While these studies suggest that e-learning is as effective as traditional educational methods, higher student satisfaction levels are indicative of more effective learning programs [75]. Therefore, the lack of longitudinal data may limit our ability to accurately evaluate the impact of e-learning technologies.

### Intervention characteristics

Many of the studies in this review used virtual patient and case-based pedagogical methods reflecting an educational trend towards more critical thinking [76]. Thirty-five of the interventions under review used an interactive approach, encouraging a style in which students collaborated and discussed ideas with their peers and tutors, the importance of which has been recognised [77]. Two studies of mobile learning identified wasted time for students as a concern that could be addressed by allowing immediate access to information that would soon be required [30, 55]. This ‘just in time learning’, defined as a “brief educational experience targeting a specific need or clinical question” [78], can be facilitated through e-learning. Ten of the included studies concluded that an integrated approach works best, whereby educators do not seek to replace traditional methods but rather supplement them. This has previously been described as a ‘blended-learning’ style [77]. A recent study suggests that students thrive in blended- versus self-directed virtual reality environments due to face-to-face teacher support [79].

### Intervention effectiveness and acceptability

Despite variability in methodological design, several studies of e-learning across domains of education, politics, business, and military training have shown knowledge gains assessed by pre- versus post-intervention tests [80]. Similarly, subjects within the studies we have reviewed have reported e-learning interventions to be conducive to learning [32, 35, 36, 44, 58, 60–62, 64, 65], have demonstrated improvements in learning [25–27, 29–31, 34, 36–39, 43, 46, 48, 49, 54–57, 66] and modified learning strategies [50, 52, 63]. The specific features of e-learning strategies most likely to enhance the learning experience may include: peer-to-peer learning [52], making use of wasted time [30, 40–42, 81], feedback from clinicians and ongoing technical support [32, 82], consolidation of information and skill through repetition [52, 82, 83], and convenience of online content access [25, 30, 40–42]. Usability of the intervention has specifically featured strongly in this review. Vital features of e-learning interventions facilitating its use may include: interactive software, active learning promotion (built-in quizzes following cases), asynchronous use, multimedia platforms (i.e., slideshows, videos, images), ease of use and adaptability [76, 81, 84]. Unsurprisingly, students are more engaged with educational material after the typical 9-to-5 work hours [25, 35]. Whereas traditional learning opportunities may be restricted to these hours, the flexibility of being able to access online resources outside of this timeframe, may better facilitate achievement of learning objectives [25, 35]. Additionally, the use of discussion boards [78] and games [77] may facilitate active learning and feedback to be sought and received in a timely manner. Furthermore, quality assurance is recognized as a critical factor, and if considered at the planning stage of an intervention and built into e-learning interventions, may lead to more favourable outcomes [23]. Engagement with students in this manner is in keeping with the AMEE recommended goals of e-learning [1]. Several studies also highlight how online learning might provide an encouraging environment for the development of knowledge and skills, relatively easily tailored to individual learning preferences and prior knowledge, and with the possibility of compensating for a lack of accessibility of patients or teachers [35, 36, 38, 63, 85]. Furthermore, the ability to access an extensive network of additional resources may allow students to take control of their learning and regulate the volume of information studied [36].

While our review found improved learning outcomes, other systematic reviews assessing the effectiveness of technology and electronic media in health education, report equivocal findings [77, 86]. Proposed factors that may limit learning capacity include: hesitancy to adopt changes by students and teachers, poor technical or financial support, limited technological skills, and the lack of direct and personalized teacher communication [25, 32, 82, 87]. For example, Davies et al. suggests that an open outlook on mobile device usage was required by students and clinicians, to limit non-use and acquire potential benefits [30]. In another study conducted by Alsoufi et al., online medical education programs implemented in Libya in response to COVID-19 were found to be negatively received by respondents [87]. Financial and technical barriers and the lack of hands-on bedside teaching were stated by respondents as limitations to acceptance of e-learning. The shift to online medical learning in the Philippines during the COVID-19 pandemic also identified lack of access to computers and the internet as a significant barrier [82]. Of course, with these later interventions, the rapid onset of the pandemic required development of e-learning platforms with relatively little training and preparation. As such, the logistics of e-learning curricula as it pertains to specific communities may not have been foreseen. Another reason for such discrepancies may be the underlying discipline in which the intervention is being evaluated [47]. For instance, the use of only e-learning materials when teaching new skills may not be sufficient, as the direct observation and guidance of an expert is valuable [88]. A blended-learning environment may be more appropriate in these circumstances [47]. Indeed, viewing e-learning as a complement rather than replacement of traditional approaches is already well accepted amongst students [80].

### Learner, preceptor and institution characteristics

The twenty-first century learners are known to be avid consumers of various digital platforms. However, studies have shown an incongruence between their ability to use technology for entertainment and ability to use it for educational purposes [89]. Most students require guidance to synthesize information and create new understanding. In fact, students in middle school through undergraduate level studies have consistently demonstrated poor digital research skills [90, 91]. Furthermore, students may require adjustment of learning practices to best engage with the presented e-learning platform. For example, use of PowerPoint presentations or handouts in replacement of in-class teaching can cause visual and auditory learners to require more time to comprehend the information [82]. Therefore, in addition to carrying an acceptant attitude and a willingness to collaborate with peers, the ability to engage with and extract relevant content from online resources, is a characteristic linked to success in e-learning [32, 66].

Nevertheless, recognition of the need for continued mentoring and support in the online learning environment, requires appreciation of the role of the e-teacher. Preceptors’ roles involve development and delivery of the intervention and acting as a resource person for the duration of the module [68]. In our previous discussion of e-learning strategy effectiveness, two further roles of the e-teacher can be recognized. Firstly, the e-teacher is instrumental in providing timely feedback, one of the main features associated with improved e-learning outcomes [32]. E-teachers should actively monitor student activity and provide feedback or support where needed [92]. Secondly, success of e-learning is also strongly related to the motivation of the students and indirectly the motivation demonstrated by the e-teacher [30, 92]. The ARCS motivational model highlights four components needed to create a highly motivational e-learning system: maintain student attention, content relevance, student confidence, student satisfaction [93]. If e-teachers can convey subject material through strategies which encompass use of interactive multimedia, humour, and inquiry for instance, they can satisfy the first component of attention [92]. Generating activities that best illustrate main ideas, tailoring to the learner knowledge level and providing positive feedback are examples of methods to instil content relevance, student confidence and student satisfaction, accordingly. In Gradl-Dietsch et al., combination of video-based learning, team-based learning and peer-teaching, along with practical skills teaching in point of care ultrasound, feedback from peer teachers, and positive instructor-learner interactions, collectively fulfil the components of the ARCS model [54]. In Sox et al., the use of a web-based module to teach oral case presentation skills satisfied student attention and content relevance [51]. However, poor adherence to module largely due to time constraints, can be suggestive of poor student satisfaction. As a result, student confidence and the quality of oral case presentations did not differ from controls (faculty-led feedback sessions). As suggested by the authors, a combination of web module with direct faculty feedback may better instil student confidence and satisfaction with module content, and thereby improve student performance [51]. Recent studies have shown that the digital literacy skills of most instructors are inadequate [90, 91]. Therefore, institutions need to invest into the provision of training programs and supports to allow e-teachers to develop and strengthen competencies needed to sufficiently handle educational technologies [92, 94, 95]. For example, the use of offline tablet-based materials was shown to improve medical education in Zambia, but reported usage amongst healthcare workers was low [95]. Authors suggest that a lack of training in tablet use was the underlying reason. Taken together, while the role of the teacher has changed compared to traditional pedological approaches, their actions can still heavily influence student learning outcomes.

### Limitations and future directions

In a field where technology is changing faster than studies can be completed and interventions are evolving rapidly, medical education research has become a challenging topic of debate. Research can “provide the evidence to prove—and improve—the quality and effectiveness of teaching” and therefore advise the restructuring of curricula to respond to advances in science and technology [96]. In this review, 29 studies received a global score of 3 or less out of 5, highlighting a lack of transparency and rigour in most of the studies. This justifies a need for a standardised approach for reporting medical education interventions. Pre- and post-intervention testing is informative, but follow-up months later would be an important measure of knowledge retention and therefore intervention effectiveness. Moreover, most of the studies in this review examined knowledge or skill development but few examined higher Kirkpatrick levels. The inclination towards focus on the lower levels of the Kirkpatrick model may stem from difficulty following students in the field to evaluate long-term results of the educational intervention on student behaviours (level three) and the organization at large (level four) [97]. Future work on the evaluation of associated changes in behaviour, professional practice or patient outcomes would be valuable. Other e-learning characteristics that can be evaluated in future work (Fig. 4) may include the capacity for adaptivity (to accommodate changing student needs and performance) and collaboration [98]. Including descriptions of curricula context can also facilitate the exploration of which e-learning strategies are best suited for specific medicine disciplines and socioeconomic settings. The use of internet resources by both students and patients alike, and the exponential growth in social media influence may also provide a platform for future e-learning interventions [99].

## Conclusions

Over the past twenty years and with the recent advent of the COVID-19 pandemic, there has been a substantial increase in the use of e-learning. This review found that e-learning interventions are positively perceived by students and associated with improvements in learning. Improved learning outcomes are closely correlated with interactive, asynchronous, easily accessible and usable interventions, and those involving students and preceptors with digital skills, high motivation and receptive attitudes. While further exploration of the strengths and weaknesses of e-learning technologies is warranted, use of online platforms is a creditable educational tool for undergraduate clinical medicine.

## Supplementary Information

**Additional file 1:** PRISMA Checklist.

**Additional file 2:  **Summary of Review Results.

**Additional file 3:  **Intervention Characteristics.

## Abbreviations

AMEE: Association for Medical Education in Europe
PRISMA: Preferred Reporting Items for Systematic Reviews and Meta-Analyses

## Declarations

### Availability of data and material

All data generated or analysed during this study are included in this published article [and its supplementary information files].

## References

[1] Ellaway R, Masters K (2008). AMEE Guide 32: e-Learning In Medical Education - Part 1: Learning. Teaching And Assessment Med Teach.

[2] Goldie JG (2016). Connectivism: A Knowledge Learning Theory For The Digital Age?. Med Teach.

[3] Maertens H, Madani A, Landry T, Vermassen F, Van Herzeele I, Aggarwal R (2016). Systematic Review Of E-Learning For Surgical Training. Br J Surg.

[4] Tarpada SP, Morris MT, Burton DA (2016). E-Learning In Orthopedic Surgery Training: A Systematic Review. J Orthop.

[5] Feng J-Y, Chang Y-T, Chang H-Y, Erdley WS, Lin C-H, Chang Y-J (2013). Systematic Review of Effectiveness of Situated E-Learning on Medical and Nursing Education. Worldviews Evid Based Nurs.

[6] Price Kerfoot B, Masser BA, Hafler JP (2005). Influence Of New Educational Technology On Problem-Based Learning At Harvard Medical School. Med Educ.

[7] Guarino S, Leopardi E, Sorrenti S, De Antoni E, Catania A, Alagaratnam S (2014). Internet-Based Versus Traditional Teaching And Learning Methods. Clin Teach.

[8] Trelease RB (2016). From Chalkboard, Slides, And Paper To E-Learning: How Computing Technologies Have Transformed Anatomical Sciences Education. Anat Sci Educ.

[9] Felder E, Fauler M, Geiler S (2013). Introducing E-Learning/Teaching In A Physiology Course For Medical Students: Acceptance By Students And Subjective Effect On Learning. Adv Physiol Educ.

[10] Martin EA (2015). Concise medical dictionary.

[11] Han H, Nelson E, Wetter N. Medical students’ online learning technology needs. Clin Teach. 2014;11(1):15–9. 10.1111/tct.12092 24405913

[12] Barton J, Rallis KS, Corrigan AE, Hubbard E, Round A, Portone G (2021). Medical students’ pattern of self-directed learning prior to and during the coronavirus disease 2019 pandemic period and its implications for Free Open Access Meducation within the United Kingdom. J Educ Eval Health Prof.

[13] O’Hanlon R, Laynor G. Responding to a new generation of proprietary study resources in medical education. J Med Libr Assoc. 2019;107(2):251–7. 10.5195/jmla.2019.619 PMC6466489 31019395

[14] Zhang J, Peterson RF, Ozolins IZ (2011). Student approaches for learning in medicine: What does it tell us about the informal curriculum?. BMC Med Educ.

[15] Cooper AL, Elnicki DM (2011). Resource utilisation patterns of third-year medical students. Clin Teach.

[16] Wynter L, Burgess A, Kalman E, Heron JE, Bleasel J (2019). Medical students: what educational resources are they using?. BMC Med Educ.

[17] Larsen DP, Butler AC, Roediger HL (2008). Test-enhanced learning in medical education. Med Educ.

[18] Augustin M (2014). How to learn effectively in medical school: test yourself, learn actively, and repeat in intervals. Yale J Biol Med.

[19] Moher D, Liberati A, Tetzlaff J, Altman DG (2009). Reprint—Preferred Reporting Items for Systematic Reviews and Meta-Analyses: The PRISMA Statement. Phys Ther.

[20] Hammond M, editor Learning From Experience: Approaching The Research Of CD-ROM In Schools. WCCE; 1995.

[21] Kentnor HE (2015). Distance Education and the Evolution of Online Learning in the United States. Curric Teach.

[22] Barry Issenberg S, McGaghie WC, Petrusa ER, Lee Gordon D, Scalese RJ (2005). Features And Uses Of High-Fidelity Medical Simulations That Lead To Effective Learning: A BEME Systematic Review. Med Teach.

[23] Evaluation. EGfGo. Guidelines for Evaluating Papers on Educational Interventions. Education Group for Guidelines on Evaluation ed: BMJ; 1999. p. 1265–7. PMC1115651 10231261

[24] Kirkpatrick D, Kirkpatrick J. Transferring Learning to Behavior : Using the Four Levels to Improve Performance. Oakland, United States: Berrett-Koehler Publishers, Incorporated; 2005.

[25] Bernardo V, Ramos MP, Plapler H, De Figueiredo LF, Nader HB, Ancao MS (2004). Web-Based Learning In Undergraduate Medical Education: Development And Assessment Of An Online Course On Experimental Surgery. Int J Med Inform.

[26] Raupach T, Munscher C, Pukrop T, Anders S, Harendza S (2010). Significant Increase In Factual Knowledge With Web-Assisted Problem-Based Learning As Part Of An Undergraduate Cardio-Respiratory Curriculum. Adv Health Sci Educ Theory Pract.

[27] Casillas JM, Gremeaux V. Evaluation Of Medical Students’ Expectations For Multimedia Teaching Materials: Illustration By An Original Method Using The Evaluation Of A Web Site On Cardiovascular Rehabilitation. Ann Phys Rehabil Med. 2012;55(1):25–37. 10.1016/j.rehab.2011.12.001 22225845

[28] Cevik AA, Shaban S, El Zubeir M, Abu-Zidan FM (2018). The Role Of Emergency Medicine Clerkship E-Portfolio To Monitor The Learning Experience Of Students In Different Settings: A Prospective Cohort Study. Int J Emerg Med.

[29] Corrigan M, Reardon M, Shields C, Redmond H. “SURGENT” – Student E-Learning For Reality: The Application Of Interactive Visual Images To Problem-Based Learning In Undergraduate Surgery. J Surg Educ. 2008;65(2):120–5. 10.1016/j.jsurg.2007.11.011 18439533

[30] Davies BS, Rafique J, Vincent TR, Fairclough J, Packer MH, Vincent R (2012). Mobile Medical Education (Momed) - How Mobile Information Resources Contribute To Learning For Undergraduate Clinical Students - A Mixed Methods Study. BMC Med Educ.

[31] de Sena DP, Fabricio DD, Lopes MH, da Silva VD (2013). Computer-Assisted Teaching Of Skin Flap Surgery: Validation Of A Mobile Platform Software For Medical Students. PLoS ONE.

[32] Howlett D, Vincent T, Gainsborough N, Fairclough J, Taylor N, Cohen J (2009). Integration Of A Case-Based Online Module Into An Undergraduate Curriculum: What Is Involved And Is It Effective?. E-Learn.

[33] Khalil R, Mansour AE, Fadda WA, Almisnid K, Aldamegh M, Al-Nafeesah A (2020). The Sudden Transition To Synchronized Online Learning During The COVID-19 Pandemic In Saudi Arabia: A Qualitative Study Exploring Medical Students’ Perspectives. BMC Med Educ.

[34] Ogura A, Hayashi N, Negishi T, Watanabe H (2018). Effectiveness of an e-Learning Platform for Image Interpretation Education of Medical Staff and Students. J Digit Imaging.

[35] Orton E, Mulhausen P (2008). E-Learning Virtual Patients For Geriatric Education. Gerontol Geriatr Educ.

[36] Sijstermans R, Jaspers MW, Bloemendaal PM, Schoonderwaldt EM (2007). Training Inter-Physician Communication Using The Dynamic Patient Simulator. Int J Med Inform.

[37] Tews M, Brennan K, Begaz T, Treat R. Medical Student Case Presentation Performance And Perception When Using Mobile Learning Technology In The Emergency Department. Med Educ Online. 2011;16. 10.3402/meo.v16i0.7327. 10.3402/meo.v16i0.7327 PMC3195427 22013378

[38] Wunschel M, Leichtle U, Wulker N, Kluba T (2010). Using A Web-Based Orthopaedic Clinic In The Curricular Teaching Of A German University Hospital: Analysis Of Learning Effect, Student Usage And Reception. Int J Med Inform.

[39] Zayed MA, Lilo EA, Lee JT (2017). Impact of an Interactive Vascular Surgery Web-Based Educational Curriculum on Surgical Trainee Knowledge and Interest. J Surg Educ.

[40] Al Zahrani EM, Al Naam YA, AlRabeeah SM, Aldossary DN, Al-Jamea LH, Woodman A (2021). E- Learning experience of the medical profession’s college students during COVID-19 pandemic in Saudi Arabia. BMC Med Educ.

[41] Dost S, Hossain A, Shehab M, Abdelwahed A, Al-Nusair L (2020). Perceptions of medical students towards online teaching during the COVID-19 pandemic: a national cross-sectional survey of 2721 UK medical students. BMJ Open.

[42] Coffey CS, MacDonald BV, Shahrvini B, Baxter SL, Lander L (2020). Student Perspectives on Remote Medical Education in Clinical Core Clerkships During the COVID-19 Pandemic. Medical Science Educator.

[43] Diekhoff T, Kainberger F, Oleaga L, Dewey M, Zimmermann E (2020). Effectiveness Of The Clinical Decision Support Tool ESR Eguide For Teaching Medical Students The Appropriate Selection Of Imaging Tests: Randomized Cross-Over Evaluation. Eur Radiol.

[44] Dombrowski T, Wrobel C, Dazert S, Volkenstein S (2018). Flipped Classroom Frameworks Improve Efficacy In Undergraduate Practical Courses – A Quasi-Randomized Pilot Study In Otorhinolaryngology. BMC Med Educ.

[45] Hari R, Kälin K, Harris M, Walter R, Serra A (2020). Comparing Blended Learning With Faculty-Led Ultrasound Training: Protocol For A Randomised Controlled Trial (The SIGNATURE Trial). Praxis (Bern 1994).

[46] Herrmann-Werner A, Weber H, Loda T, Keifenheim KE, Erschens R, Mölbert SC, et al. “But Dr Google Said…” - Training Medical Students How To Communicate With E-Patients. Med Teach. 2019;41(12):1434–40. 10.1080/0142159X.2018.1555639 30707847

[47] Jenkins S, Goel R, Morrell DS (2008). Computer-Assisted Instruction Versus Traditional Lecture For Medical Student Teaching Of Dermatology Morphology: A Randomized Control Trial. J Am Acad Dermatol.

[48] Lee LA, Chao YP, Huang CG, Fang JT, Wang SL, Chuang CK (2018). Cognitive Style and Mobile E-Learning in Emergent Otorhinolaryngology-Head and Neck Surgery Disorders for Millennial Undergraduate Medical Students: Randomized Controlled Trial. J Med Internet Res.

[49] Plackett R, Kassianos AP, Kambouri M, Kay N, Mylan S, Hopwood J (2020). Online Patient Simulation Training To Improve Clinical Reasoning: A Feasibility Randomised Controlled Trial. BMC Med Educ.

[50] Schneider AT, Albers P, Muller-Mattheis V (2015). E-Learning in Urology: Implementation of the Learning and Teaching Platform CASUS(R) - Do Virtual Patients Lead to Improved Learning Outcomes? A Randomized Study among Students. Urol Int.

[51] Sox CM, Tenney-Soeiro R, Lewin LO, Ronan J, Brown M, King M (2018). Efficacy of a Web-Based Oral Case Presentation Instruction Module: Multicenter Randomized Controlled Trial. Acad Pediatr.

[52] Sward KARNP, Richardson SRNP, Kendrick JMD, Maloney CMDP (2008). Use of a Web-Based Game to Teach Pediatric Content to Medical Students. Acad Pediatr.

[53] Wahlgren CF, Edelbring S, Fors U, Hindbeck H, Stahle M (2006). Evaluation Of An Interactive Case Simulation System In Dermatology And Venereology For Medical Students. BMC Med Educ.

[54] Gradl-Dietsch G, Menon AK, Gürsel A, Götzenich A, Hatam N, Aljalloud A (2018). Basic Echocardiography For Undergraduate Students: A Comparison Of Different Peer-Teaching Approaches. Eur J Trauma Emerg Surg.

[55] Davis JS, Garcia GD, Wyckoff MM, Alsafran S, Graygo JM, Withum KF (2012). Use Of Mobile Learning Module Improves Skills In Chest Tube Insertion. J Surg Res.

[56] Roesch A, Gruber H, Hawelka B, Hamm H, Arnold N, Popal H, et al. Computer Assisted Learning In Medicine: A Long-Term Evaluation Of The “Practical Training Programme Dermatology 2000.” Med Inform Internet Med. 2003;28(3):147–59. 10.1080/14639230310001613430 14612304

[57] Sendra-Portero F, Torales-Chaparro OE, Ruiz-Gomez MJ, Martinez-Morillo M (2013). A Pilot Study To Evaluate The Use Of Virtual Lectures For Undergraduate Radiology Teaching. Eur J Radiol.

[58] Farrimond H, Dornan TL, Cockcroft A, Rhodes LE (2006). Development And Evaluation Of An E-Learning Package For Teaching Skin Examination. Action Research Br J Dermatol.

[59] Moriates C, Valencia V, Stamets S, Joo J, MacClements J, Wilkerson L (2019). Using Interactive Learning Modules to Teach Value-Based Health Care to Health Professions Trainees Across the United States. Acad Med.

[60] Naeger DM, Straus CM, Phelps A, Courtier J, Webb EM (2014). Student-Created Independent Learning Modules: An Easy High-Value Addition To Radiology Clerkships. Acad Radiol.

[61] Smith E, Boscak A (2021). A Virtual Emergency: Learning Lessons From Remote Medical Student Education During The COVID-19 Pandemic. Emerg Radiol.

[62] Taurines R, Radtke F, Romanos M, König S (2020). Using Real Patients In E-Learning: Case-Based Online Training In Child And Adolescent Psychiatry. GMS J Med Educ.

[63] De Villiers M, Walsh S (2015). How Podcasts Influence Medical Students’ Learning – A Descriptive Qualitative Study. Afr J Health Prof Educ.

[64] Wagner-Menghin M, Szenes V, Scharitzer M, Pokieser P (2020). Designing Virtual Patient Based Self-Study Quizzes Covering Learning Goals In Clinical Diagnostic Sciences For Undergraduate Medical Students - The Radiology Example. GMS J Med.

[65] Nelson TM (2018). Preparing for Practice: Strengthening Third-Year Medical Students’ Awareness of Point-of-Care Resources. Med Ref Serv Q.

[66] Kourdioukova EV, Verstraete KL, Valcke M (2011). The Quality And Impact Of Computer Supported Collaborative Learning (CSCL) In Radiology Case-Based Learning. Eur J Radiol.

[67] Clark RC, Mayer RE. E-Learning and the Science of Instruction: Proven Guidelines for Consumers and Designers of Multimedia Learning. Hoboken: Center for Creative Leadership; 2016.

[68] Gruner D, Pottie K, Archibald D, Allison J, Sabourin V, Belcaid I (2015). Introducing global health into the undergraduate medical school curriculum using an e-learning program: a mixed method pilot study. BMC Med Educ.

[69] UNESCO. National education responses to COVID-19: summary report of UNESCO's online survey. United Nations Educational, Scientific and Cultural Organization.

[70] Mann KV (2011). Theoretical Perspectives In Medical Education: Past Experience And Future Possibilities. Med Ed.

[71] Moreno R, Mayer RE (1999). Cognitive Principles Of Multimedia Learning: The Role Of Modality And Contiguity. J Educ Psychol.

[72] Kim S (2006). The Future of e-Learning in Medical Education: Current Trend and Future Opportunity. J Educ Eval Health Prof.

[73] Du S, Liu Z, Liu S, Yin H, Xu G, Zhang H (2013). Web-based distance learning for nurse education: a systematic review. Int Nurs Rev.

[74] Lahti M, Hätönen H, Välimäki M (2014). Impact of e-learning on nurses’ and student nurses knowledge, skills, and satisfaction: A systematic review and meta-analysis. Int J Nurs Stud.

[75] Kirkpatrick DL, Brown SM, Seidner CJ (1998). The Four Levels of Evaluation. Evaluating Corporate Training: Models and Issues.

[76] Fuks A, Boudreau JD, Cassell EJ (2009). Teaching Clinical Thinking To First-Year Medical Students. Med Teach.

[77] Chumley-Jones HS, Dobbie A, Alford CL (2002). Web-Based Learning: Sound Educational Method Or Hype? A Review Of The Evaluation Literature. Acad Med.

[78] Kahn CE Jr, Ehlers KC, Wood BP. Radiologists’ Preferences for Just-in-Time Learning. J Digit Imaging. 2006;19(3):202–6. 10.1007/s10278-005-9242-y PMC3045145 16680513

[79] Fairén M, Moyés J, Insa E (2020). VR4Health: Personalized teaching and learning anatomy using VR. J Med Syst.

[80] Ruiz JG, Mintzer MJ, Leipzig RM (2006). The impact of E-learning in medical education. Acad Med.

[81] Klímová B (2018). Mobile Learning in Medical Education. J Med Syst.

[82] Baticulon RE, Sy JJ, Alberto NRI, Baron MBC, Mabulay REC, Rizada LGT (2021). Barriers to Online Learning in the Time of COVID-19: A National Survey of Medical Students in the Philippines. Medical Science Educator.

[83] Mehrpour SR, Aghamirsalim M, Motamedi SM, Ardeshir Larijani F, Sorbi R (2013). A supplemental video teaching tool enhances splinting skills. Clin Orthop Relat Res.

[84] Larvin M (2009). E-Learning In Surgical Education And Training. ANZ J Surg.

[85] Lane J, Slavin S. Simulation in Medical Education: A Review. Simulation & Gaming - Simulat Gaming. 2001;32.

[86] Bell DS, Fonarow GC, Hays RD, Mangione CM (2000). elf-study from web-based and printed guideline materials. A randomized, controlled trial among resident physicians. Annals of internal medicine.

[87] Alsoufi A, Alsuyihili A, Msherghi A, Elhadi A, Atiyah H, Ashini A, et al. Impact of the COVID-19 pandemic on medical education: Medical students’ knowledge, attitudes, and practices regarding electronic learning. PLoS ONE. 2020;15(11):e0242905. 10.1371/journal.pone.0242905 PMC7688124 33237962

[88] Rogers DA, Regehr G, Yeh KA, Howdieshell TR (1998). Computer-assisted Learning versus a Lecture and Feedback Seminar for Teaching a Basic Surgical Technical Skill. The American journal of surgery.

[89] Guri RS (2018). E-Teaching In Higher Education: An Essential Prerequisite For E-Learning. J New Approaches Educ Res.

[90] Alexander B, Adams-Becker S, Cummins M, Hall-Giesinger C. Digital Literacy in Higher Education, Part II: An NMC Horizon Project Strategic Brief. . Austin, Texas; 2017.

[91] Wineburg S, McGrew S, Breakstone J, Ortega T. Evaluating Information: The Cornerstone of Civic Online Reasoning. Stanford Digital Repository; 2016.

[92] Yengin İ, Karahoca D, Karahoca A, Yücel A (2010). Roles Of Teachers In E-Learning: How To Engage Students & How To Get Free E-Learning And The Future. Procedia Soc.

[93] Keller J, Suzuki K (2004). Learner Motivation and E-learning Design: A Multinationally Validated Process. Learn Media Technol.

[94] Howe DL, Heitner KL, Dozier A, Silas S (2021). Health Professions Faculty Experiences Teaching Online During the COVID-19 Pandemic. ABNF J.

[95] Barteit S, Neuhann F, Bärnighausen T, Bowa A, Lüders S, Malunga G (2019). Perspectives of Nonphysician Clinical Students and Medical Lecturers on Tablet-Based Health Care Practice Support for Medical Education in Zambia, Africa: Qualitative Study. JMIR Mhealth Uhealth.

[96] Easton G (2014). Primary care education research-time to raise our game?. Educ Prim Care.

[97] Cahapay M (2021). Kirkpatrick Model: Its Limitations As Used in Higher Education Evaluation. IJATE.

[98] Byrnes KG, Kiely PA, Dunne CP, McDermott KW, Coffey JC. Communication, collaboration and contagion: “Virtualisation” of anatomy during COVID-19. Clin Anat. 2021;34(1):82–9. 10.1002/ca.23649 PMC7404681 32648289

[99] Dunne S, Cummins NM, Hannigan A, Shannon, Dunne C, Cullen W (2013). A method for the design and development of medical or health care information websites to optimize search engine results page rankings on Google. Journal of Medical Internet Research.

