# Supplementary material for: Advances in e-learning in undergraduate clinical medicine: a systematic review
Source: BMC Med Educ. 2022 Oct 7;22:711. doi: 10.1186/s12909-022-03773-1 (PMC9540295; doi:10.1186/s12909-022-03773-1)
Supplement: Supplementary file 2 — Additional file 2: Summary of Review Results. [file 12909_2022_3773_MOESM2_ESM.docx]

**Additional File 2: Summary of Review Results**

| **Author, Year** | **Context** | **Intervention** | **Learning Theory** | **Cognitive Domain** | **Accessibility factors** | **Collaborative factors** | **Evaluation method** | **Global Score^b^** | **Kirkpatrick’s Level^a^** | **Usability** | **Preceptor Role** |
| --- | --- | --- | --- | --- | --- | --- | --- | --- | --- | --- | --- |
| Casillas, 2012 [27] | Not reported | Multimedia: audio and video  Slideshow  Didactic | Not reported | Knowledge | Synchronous | Not assessed | Pre- and post- MCQ (immediately after)  Student survey | 2 | 2 | Not assessed | Not assessed |
| Corrigan, 2008 [29] | Integration into existing curriculum | PBL cases, online case simulation  Images  Interactive | Constructivism | Knowledge | Asynchronous | Not assessed | Final exam results  Student survey | 2.5 | 2 | Not assessed | Not assessed |
| Howlett, 2009 [32] | Integration into existing curriculum | Multimedia: Images, video  PBL cases, online cases  Interactive | Not reported | Knowledge | Asynchronous | Not assessed | Student survey | 2.5 | 1 | Not assessed | Write cases  Assess questionnaires  IT Support |
| Davies, 2012 [30] | Not reported | Mobile information  Interactive | Cognitivism | Knowledge | Asynchronous | Not assessed | Student survey | 4 | 2 | Assessed students for opinions on visual display adaptability | Introductory lectures  Ongoing support |
| Farrimond, 2006 [58] | Not reported | Multimedia: online interactive tutorial, images | Constructivism | Knowledge | Synchronous | None | Student survey | 3 | 1 | Assessed by Software Users Measurement Inventory | Not assessed |
| De Villiers, 2015 [63] | Not reported | Podcasts: audio, video, text  Didactic | Not reported | Knowledge | Asynchronous | None | Student survey | 2.5 | 3 | Not assessed | Not assessed |
| Davis, 2012 [55] | Not reported | Multimedia: voice, soundtrack, live animation, and video  Didactic | Not reported | Knowledge | Synchronous | None | Observation of technique | 2 | 2 | Not assessed | Supervision |
| Orton, 2008 [35] | Not reported | Virtual patients  Case based simulation  Interactive | Not reported | Knowledge | Synchronous | Students question and diagnose the patient in tutorial groups | Student survey | 2 | 1 | Not assessed | Not assessed |
| Naeger, 2014 [60] | Fills gaps identified in the curriculum | Multimedia: images, quiz  Interactive  Students created online learning modules | Not reported | Knowledge | Asynchronous | Not included | Student survey | 3 | 1 | Not assessed | Goal setting  Evaluations  Moderating discussion |
| Kourdio-ukova, 2011 [66] | Not reported | PBL cases  Online MSK radiology tutorial cases  Interactive | Not reported | Knowledge | Asynchronous | Cases were resolved by group discussion | Student survey  Quantitative analysis of discussions  Final exam scores | 3 | 2 | Not assessed | Set training facility  Analyse questionnaires  Track student participation |
| Wunschel, 2010 [38] | Integration into existing curriculum | Multimedia: images and video  Virtual patients  Interactive | Not reported | Knowledge and Skills | Asynchronous | Not assessed | Student survey  Pre- and post- intervention scores (immediately after) | 2.5 | 2 | Not assessed | Not assessed |
| Wahlgren, 2006 [53] | Not reported | Multimedia: images and video  PBL cases  Virtual patients  Interactive | Not reported | Knowledge | Synchronous | Not assessed | Student survey  Pre- and post- intervention scores (immediately after) | 3.5 | 2 | Assessed students for opinions on layout, user friendliness and clarity | Oversee intervention |
| Sijstermans, 2007 [36] | Not reported | Multimedia: images and video  Virtual patients  Interactive | Not reported | Skills | Asynchronous | Students were paired and together were responsible for managing their patient | Pre- and post-intervention scores (immediately after)  Self-assessment questionnaire | 3 | 2 | Assessed students for opinions on navigation, layout and structure | Analyse results, log times and questionnaires |
| Sendra-Portero, 2013 [57] | Not reported | Multimedia: audio and video  Online lecture  Didactic | Not reported | Knowledge | Asynchronous | Not assessed | Post intervention scores (immediately after) | 2.5 | 2 | Design and content were assessed by students | Lectures  Evaluate questionnaires |
| Schneider, 2015 [50] | Not reported | Virtual patients  Interactive  Feedback provided by consultants at the end | Not reported | Knowledge | Asynchronous | Not assessed | Post intervention scores (immediately after) | 3 | 3 | Not assessed | Create virtual patients |
| Raupach, 2010 [26] | ‘Blended’ into existing curriculum | Virtual PBL cases  Interactive | Not reported | Knowledge | Asynchronous | Online group discussion | Student survey  Final course assessment | 3 | 2 | Not assessed | Ongoing support Evaluations |
| Roesch, 2003 [56] | Integration into existing curriculum | Multimedia: images, videos  Virtual patients  Feedback from virtual doctors  Interactive | Cognitivism | Knowledge and Skills | Asynchronous | None | Student survey (3 mo after)  Post- intervention MCQ (immediately after) | 2.5 | 2 | Instructional design, ergonomics and presentation were assessed by student questionnaire | Not assessed |
| Bernardo, 2004 [25] | Supplement to current curriculum | Multimedia: images, quizzes  Online discussion  Interactive | Not reported | Knowledge | Asynchronous | Collaborative activities through discussion board | Student survey  Pre and post- intervention scores   (immediately after) | 2.5 | 2 | Not assessed | Answer questions  Ongoing support  Design quizzes  Facilitate discussion |
| Jenkins, 2008 [47] | Not reported | Multimedia: audio and images  Interactive | Not reported | Knowledge | Not reported | Not included | Post intervention scores (immediately after) | 3 | 2 | Not assessed | Not assessed |
| Sward, 2008 [52] | Not reported | Video game flash cards  Interactive | Constructivism | Knowledge | Synchronous | Students played the game in groups | Pre- and post-intervention scores (immediately and after 6wks) | 3 | 3 | Not assessed | Not assessed |
| Diekhoff, 2020 [43] | Supplementary resource | Clinical decision support tool (ESR eGUIDE)  Interactive | Not reported | Knowledge | Synchronous | Not assessed | Random cross-over case scenario evaluation | 3 | 2 | Not assessed | Not assessed |
| Gradl-Dietsch, 2018 [54] | New course involving peer-teaching environment | Video-based learning  Interactive | Not reported | Knowledge | Asynchronous | Not assessed | Student survey  Pre- and post- MCQ (after 2 wks)  OSPE exam | 4 | 2 | Not assessed | Create instructional videos  Provide technical support and feedback |
| Ogura, 2018 [34] | Not reported | e-learning system software used for the interpretation of radiograms  Interactive | Not reported | Knowledge | Synchronous | Not assessed | Pre- and post-intervention scores (1 wk and 2 wks after) | 2.5 | 2 | Not assessed | Not assessed |
| Sox, 2018 [51] | Integration into existing curriculum | Web-based oral case presentation  instruction module  Interactive | Constructivism | Skills | Synchronous | Not assessed | Faculty-rated quality of students’ presentations and self-reported performance | 4 | 2 | Not assessed | Student evaluation  Provide feedback |
| Lee, 2018 [48] | Evaluation of cognitive style and use of existing e-learning curriculum | Game-based quizzes Multimedia situational tests Interactive | Not reported | Knowledge | Asynchronous | Not assessed | Student survey  Pre- and post- MCQ (immediately after)  Cognitive style assessed with 25-item GEFT | 4 | 2 | Not assessed | Not assessed |
| Cevik, 2018 [28] | Clerkship curriculum development | e-portfolio  Interactive | Not reported | Knowledge | Asynchronous | Not assessed | Direct observation | 3.5 | 2 | Not assessed | Provide feedback  Accept, modify, or cancel the logbook entries according to their own judgment  Evaluate clinical performance at end of clinical shifts |
| Khalil, 2020 [33] | Curriculum replacement due to COVID | Lectures, case discussions, 4-box case analysis, clinical case discussions, online seminars, and dry labs (online laboratory demonstrations)  Interactive | Behaviorist | Knowledge | Synchronous | Not assessed | Virtual focus group discussions | 4 | 2 | Not assessed | Focus group moderation |
| Smith, 2021 [61] | Curriculum replacement due to COVID | Virtual elective  Flipped classroom  Interactive | Not reported | Knowledge | Asynchronous | Weekly interactive sessions, engaging with instructors and other students | Pre- and post-survey (immediately after) | 4 | 1 | Not assessed | Facilitate case review sessions, guiding students through select imaging cases |
| Moriates, 2019 [59] | Integration into existing curriculum | Interactive online modules | Cognitivism | Knowledge and Skills | Asynchronous | Facilitated peer discussions | Student survey | 3 | 3 | Not assessed | Not assessed |
| Nelson, 2018 [65] | Integration into existing curriculum | Online modules  Didactic | Not reported | Knowledge and Skills | Asynchronous | Not assessed | Anonymous course evaluation | 2.5 | 1 | Easy to navigate | Not assessed |
| Dombrowski, 2018 [44] | In conjunction with curriculum | Moodle e-learning environment (clinical examination videos, guided clinical cases, quizzes on visual diagnoses or tests)  Interactive | Not reported | Knowledge and Skills | Asynchronous | Not assessed | Student questionnaire | 4 | 1 | Very good evaluation of stability, speed, ease of use, quality, and topicality | Not assessed |
| Plackett, 2020 [49] | ‘Blended’ into existing curriculum | Electronic Clinical Reasoning Educational Simulation Tool (eCREST)  Interactive | Constructivism | Skills | Asynchronous | Not assessed | At 1 week- self reported survey for clinical reasoning and acceptability  At 1 month- self reported clinical reasoning survey, observed clinical reasoning measure via additional case and knowledge quiz | 4 | 2 | Not assessed | Evaluate student clinical reasoning |
| Hari, 2020 [45] | New blended-learning course | Examination videos  Interactive | Not reported | Knowledge and Skills | Asynchronous | Not assessed | On-line questionnaire; 6-station practical ultrasound exam (OSCE 1) immediately after and at 6 months | 2.5 | 2 | Not assessed | Assessed practical exam |
| Herrmann-Werner, 2019 [46] | New blended-learning course | Stimulated patients  Interactive | Not reported | Skills | Asynchronous | Engaged in role-plays | Direct observation  Self-evaluation | 3 | 2 | Not assessed | Demonstrate patient encounter  Facilitate interactive discussions  Student evaluation |
| Zayed, 2017 [39] | Supplementary resource | Web-based modules  Interactive | Not reported | Knowledge | Asynchronous | Not assessed | Student survey  Pre- and post-intervention scores (immediately after) | 3 | 2 | Not assessed | Not assessed |
| Taurines, 2020 [62] | Curriculum replacement due to COVID | Case-based online training (including stimulated patients)  Interactive | Not reported | Knowledge | Asynchronous | Not assessed | Student survey | 2.5 | 1 | Not assessed | Revise patient case  Provide information on the patient’s further development and psycho-/pharmacotherapy |
| Wagner-Menghin, 2020 [64] | Curriculum replacement due to COVID | Virtual patient-based quizzes and webinars  Interactive | Not reported | Knowledge | Asynchronous | Not assessed | Self-study quizzes | 2 | 1 | Not assessed | Not assessed |
| Tews, 2011 [37] | Just-in-time learning | Instructional videos  Didactic | Not reported | Skills | Synchronous | Not assessed | Post-study survey. | 4 | 2 | Students preferred using videos to review information instead of reading it | Fill data collection sheet (clinical evaluation)  Provide feedback |
| de Sena, 2013 [31] | Supplementary resource | Multimedia computer- assisted learning tool  Interactive | Not reported | Skills | Synchronous | Not assessed | Direct observation  Pre- and post-intervention scores(immediately after) | 4 | 2 | Not assessed | Complete checklist and a global performance assessment |
| Al Zahrani, 2021 [40] | Curriculum replacement due to COVID | e-learning platform (Blackboard Collaborate, ZOOM)  Interactive | Not reported | Knowledge | Asynchronous | Online course discussions with instructor and other students | Student questionnaire | 2 | 2 | Assessed students for opinions on accessibility, health and safety, cost-effectiveness, and learning environment | Student feedback and evaluation |
| Dost, 2020 [41] | Curriculum replacement due to COVID | Video tutorials, online question banks, pre-recorded and live tutorials, and online flashcards  Interactive | Not reported | Knowledge and Skills | Asynchronous | Interact via the chat box or by directly speaking to lecturer, group discussions, online case simulations | Student questionnaire | 2.5 | 2 | Assessed students for opinions on accessibility, cost-effectiveness, and learning environment | Student tutorials |
| Coffey, 2020 [42] | Curriculum replacement due to COVID | Case conferences, journal clubs, case simulations (such as Aquifer; Lebanon, NH, USA), and online question banks (such as UWorld; Dallas, TX, USA)  Interactive | Not reported | Knowledge | Both | Interactive remote sessions | Student questionnaire | 4 | 2 | Assessed students for opinions on accessibility, health and safety, and learning environment | Student feedback, didactic lectures, evaluations/progress |

^a^Kirkpatrick’s levels [14]: Level 1, learner’s reaction; Level 2, learning, change in knowledge, skills or experience; Level 3, behavioural change; Level 4, overall impact on the organization.. ^b^Global score (marked out of five) assigned based on Guidelines for Evaluating Papers on Medical Education Interventions from the Education Group for Guidelines on Evaluation [13].
