# Supplementary material for: Advances in e-learning in undergraduate clinical medicine: a systematic review
Source: BMC Med Educ. 2022 Oct 7;22:711. doi: 10.1186/s12909-022-03773-1 (PMC9540295; doi:10.1186/s12909-022-03773-1)
Supplement: Supplementary file 3 — Additional file 3: Intervention Characteristics. [file 12909_2022_3773_MOESM3_ESM.docx]

**Additional File 3: Intervention Characteristics**

| Author, Year | Country | Discipline | Study design | Number of participants | Year of medical school |
| --- | --- | --- | --- | --- | --- |
| Casillas, 2012 [27] | France | Cardiovascular rehabilitation | Non-comparative | 18 | 4, 5, 6 |
| Corrigan, 2008 [29] | Ireland | Surgery | Compared results with previous final year results | 115 | Final |
| Howlett, 2009 [32] | United Kingdom | Inter-disciplinary* | Non-comparative | 101 | 5 |
| Davies, 2012 [30] | United Kingdom | Inter-disciplinary* | Non-comparative | 387 | 3, 4, 5 |
| Farrimond, 2006 [58] | United Kingdom | Dermatology | Non-comparative | 13 | 4 |
| De Villiers, 2015 [63] | South Africa | Inter-disciplinary* | Non-comparative | 38 (clinical year students not specified) | 1-6 |
| Davis, 2012 [55] | United States | Surgery | Comparative | 21 in intervention group  21 in control group (did not view the mobile learning module or receive any other instruction) | 3 and above |
| Orton, 2008 [35] | United States | Geriatric medicine | Non-comparative | 120 | 3 |
| Naeger, 2014 [60] | United States | Radiology | Non-comparative | 64 | ‘Senior’ years |
| Kourdio-ukova, 2011 [66] | Belgium | Radiology | Non-comparative | 233 | 5, 7 |
| Wunschel, 2010 [38] | Germany | Orthopaedic surgery | Non-comparative | 160 | 5 |
| Wahlgren, 2006 [53] | Sweden | Dermatology and Venereology | Comparative | 85 in intervention group  31 in control group (conventional teaching (i.e. lectures, seminars/workshops, bed-side teaching, out- patient clinics) | Not reported |
| Sijstermans, 2007 [36] | The Netherlands | Inter-disciplinary* | Non-comparative | 134 | 5 |
| Sendra-Portero, 2013 [57] | Spain | Radiology | Comparative | 89 in intervention group 102 non- participants used as control (conventional lectures) | 3 |
| Schneider, 2015 [50] | Germany | Urology | Comparative | 33 in intervention group  28 in control group (textbooks to prepare the topic) | 4 |
| Raupach, 2010 [26] | Germany | Cardio-respiratory medicine | Comparative | 40 in intervention  34 in control (traditional cardio-respiratory curriculum) | 4 |
| Roesch, 2003 [56] | Germany | Dermatology | Comparative | 30 in intervention  7 in control (conventional apprenticeship) |  |
| Bernardo, 2004 [25] | Brazil | Surgery | Non-comparative | 56 | 3 |
| Jenkins, 2008 [47] | United States | Dermatology | Comparative | 36 in intervention  37 in control (traditional lecture) | 2 |
| Sward, 2008 [52] | United States | Paediatrics | Comparative | 52 in intervention group  48 in control group (self-study reviewing flash cards, classroom teaching) | 3 |
| Diekhoff, 2020 [43] | Germany | Radiology | Comparative | 40 | Not reported |
| Gradl-Dietsch, 2018 [54] | Germany | Cardiology | Comparative | 21 in control (peer teaching)  17 in peer teaching using Peyton’s four-step approach  18 in team based learning  23 in video-based learning | 2 |
| Ogura, 2018 [34] | Japan | Radiology | Comparative | 10 | 3 |
| Sox, 2018 [51] | United States | Paediatrics | Comparative | 256 in online intervention  263 in feedback intervention  270 in control (attended a lecture on delivering oral case presentations in pediatrics) | Not reported |
| Lee, 2018 [48] | Taiwan | Otorhinolaryngology-Head And Neck Surgery | Comparative | 30 in intervention  30 in control (conventional Microsoft PowerPoint instructional materials) | Not reported |
| Cevik, 2018 [28] | United Arab Emirates | Emergency Medicine | Comparative | 76 | Final |
| Khalil, 2020 [33] | Saudi Arabia | Inter-disciplinary* | Non-Comparative | 60 (clinical year students not specified) | 1^st^ year to Final |
| Smith, 2021 [61] | United States | Emergency Radiology | Comparative | 13 | 3, 4 |
| Moriates, 2019 [59] | United States | Inter-disciplinary* | Non-Comparative | 50 | 2 |
| Nelson, 2018 [65] | United States | Inter-disciplinary* | Noncomparative | 149 | 3 |
| Dombrowski, 2018 [44] | Germany | Otorhinolaryngology-Head And Neck Surgery | Comparative | 109 in intervention  103 in control (practical course) | 5 |
| Plackett, 2020 [49] | United Kingdom | Inter-disciplinary* | Comparative | 137 in intervention  127 in control (traditional teaching) | Final |
| Hari, 2020 [45] | Switzerland | Inter-disciplinary* | Comparative | 76 in intervention  76 in control (faculty led course) | 2, 3, 4 |
| Herrmann-Werner, 2019 [46] | Germany | Inter-disciplinary* | Comparative | 46 | 5, 6 |
| Zayed, 2017 [39] | United States | Vascular Surgery | Comparative | 53 trainees (36 medical students and 16 surgical residents)  31 in intervention  21 in control (highly encouraged throughout the study period to read, study, attend weekly educational conferences, participate in daily surgical rounds, and attend surgical procedures in the operating room ) | Senior |
| Taurines, 2020 [62] | Germany | Child and Adolescent Psychiatry | Noncomparative | Not reported | 5 |
| Wagner-Menghin, 2020 [64] | Austria | Radiology | Noncomparative | 486 | 5 |
| Tews, 2011 [37] | United States | Emergency | Comparative | 22 | 4 |
| de Sena, 2013 [31] | United States | Plastic Surgery | Comparative | 25 in intervention  25 in control (text-based print article) | 5,6 |
| Al Zahrani, 2021 [40] | Saudi Arabia | Inter-disciplinary* | Noncomparative | 240 (clinical year students not specified) | 1-7 |
| Dost, 2020 [41] | United Kingdom | Inter-disciplinary* | Noncomparative | 42190 (clinical year students not specified) | All years (years 1 to 5 and intercalated year) |
| Coffey, 2020 [42] | United States | Inter-disciplinary* | Noncomparative | 96 | 3 |

*Interdisciplinary studies involve interventions assessed in multiple clinical settings including medicine, neurology, gynaecology, paediatrics, psychiatry, and surgery.
